# Comparison of osteoclast differentiation protocols from human induced pluripotent stem cells of different tissue origins

**DOI:** 10.21203/rs.3.rs-3089289/v1

**Published:** 2023-07-07

**Authors:** Alexander Blümke, Erica Ijeoma, Jessica Simon, Rachel Wellington, Medania Purwaningrum, Sergei Doulatov, Elizabeth Leber, Marta Scatena, Cecilia M Giachelli

**Affiliations:** University of Washington Department of Bioengineering; University of Washington Department of Bioengineering; University of Washington Department of Bioengineering; University of Washington Department of Bioengineering; University of Washington Department of Bioengineering; University of Washington; University of Washington Department of Bioengineering; University of Washington Department of Bioengineering; University of Washington Department of Bioengineering

**Keywords:** Human induced pluripotent stem cells, osteoclasts, osteoclastogenesis, hematopoietic differentiation, mesodermal differentiation, mineral resorption

## Abstract

**Background:**

Ever since their discovery, induced pluripotent stem cells (iPSCs) have been extensively differentiated into a large variety of cell types. However, a limited amount of work has been dedicated to differentiating iPSCs into osteoclasts. While several differentiation protocols have been published, it remains unclear which protocols or differentiation methods are preferrable regarding the differentiation of osteoclasts.

**Methods:**

In this study we compare the osteoclastogenesis capacity of a peripheral blood mononuclear cell (PBMC)-derived iPSC line to a fibroblast-derived iPSC line in conjunction with either embryoid body-based or monolayer-based differentiation strategies. Both cell lines and differentiation protocols were investigated regarding their ability to generate osteoclasts and their inherent robustness and ease of use. The ability of both cell lines to remain undifferentiated while propagating using a feeder-free system was assessed using alkaline phosphatase staining. This was followed by evaluating mesodermal differentiation and the characterization of hematopoietic progenitor cells using flow cytometry. Finally, osteoclast yield and functionality based on resorptive activity, Cathepsin K and tartrate-resistant acid phosphatase (TRAP) expression were assessed. Results were validated using qRT-PCR throughout the differentiation stages.

**Results:**

Embryoid-body based differentiation yielded CD45^+^, CD14^+^, CD11b^+^ subpopulations which in turn differentiated into osteoclasts which demonstrated TRAP positivity, Cathepsin K expression and mineral resorptive capabilities. This was regardless of which iPSC line was used. Monolayer-based differentiation yielded lower quantities of hematopoietic cells that were mostly CD34^+^ and did not subsequently differentiate into osteoclasts.

**Conclusions:**

The outcome of this study demonstrates the successful differentiation of osteoclasts from iPSCs in conjunction with the embryoid-based differentiation method, while the monolayer-based method did not yield osteoclasts. No differences were observed regarding osteoclast differentiation between the PBMC and fibroblast-derived iPSC lines.

## Background

Ever since the discovery of resetting somatic cells to an embryonic-like state by Shinya Yamanaka in 2006 (1), induced pluripotent stem cells (iPSCs) have opened up previously inconceivable possibilities in the area of regenerative medicine, disease modeling and drug discovery (2). As was later established, the creation of these cells by transfecting them with the so-called Yamanaka factors (Oct3/4, Sox2, Klf4, c-Myc) allowed for their differentiation into all three germ-layers and subsequently into numerous terminally differentiated cell types (3–10), thereby proving their pluripotency. As such, iPSCs provide the possibility to create any conceivable cell type to be used in an autologous manner (11, 12). Even though challenges and considerations still exist when it comes to clinical translation (11), the possibility for autologous usage could allow for the treatment of currently untreatable diseases (13–15).

One such scenario of autologous usage of iPSCs consists in the utilization of engineered osteoclasts (OCs) as a strategy to treat bone diseases (16) and disorders involving ectopic calcifications (17–19). Our group previously developed engineered murine OCs (iRANK cells) by transfecting OC precursors with a viral construct containing an intracellular receptor activator of nuclear κB (RANK) signaling domain linked to a fusion protein (FKBP12) that provides a binding site for the chemical inducer of dimerization (CID), allowing for a drug-controlled cell differentiation independent of receptor activator of nuclear factor κB ligand (RANKL) and osteoprotegerin (OPG) (17, 20). These engineered iRANK cells haven been shown to be effective in applications involving the controlled resorption of heterotopic ossifications (17, 21), necrotic bone (22) and even more potential applications can be envisioned. Additionally, iRANK cells have also been used in disease modeling of diseases that involve OCs (22).

In terms of transitioning from murine cell to human cells, a robust source for human OCs is necessary not only for the creation of human iRANK cells but also other applications that require human OCs (23, 24). While CD34^+^ peripheral blood mononuclear cells (PBMCs) are still used as the prevalent source for OCs by many (25–27), the small number that can be harvested from a patient in conjunction with the inability to expand them *in vitro* (28, 29) limit their potential autologous use. iPSCs on the other hand can overcome the problem of cell number limitation as they can be expanded *in vitro* indefinitely and thereby allow for upscaling of OC production. Additionally, iPSCs can be created from a wide variety of tissue origins and do not require large amounts of blood to be drawn from the patient.

Several iPSC lines have to date been successfully differentiated into OCs (30, 31). However, the entire process of generating human OCs from iPSCs can differ widely (32). Differences may arise starting with the vector for iPSC creation (33–35) and the protocols used for iPSC expansion (36). In order to generate OCs, iPSCs must undergo mesodermal and hematopoietic differentiation, which is followed by terminal OC differentiation (10, 37–39). Different approaches for mesodermal and hematopoietic differentiation have been published. One approach involves the creation of a single-cell iPSC suspension which is used to created small, spherical, embryoid body-like structures, which are said to simulate early stages of post-implantation embryonic development (4, 40). Another approach uses iPSC colonies in monolayer to start mesodermal induction and hematopoietic differentiation (41, 42). Both approaches give rise to cell-forming complexes that produce hematopoietic progenitor cells (HPCs) but differ in the cytokines used for differentiation. While several differentiation protocols have been published, it remains unclear which protocols or differentiation methods are preferrable regarding the efficient and robust differentiation of human OCs.

In this study, we compare a PBMC-derived iPSC line to a fibroblast-derived iPSC line (PBMC-derived vs. fibroblast-derived) in conjunction with either an embryoid body-based (EB) (31) or a monolayer-based (MB) (37) differentiation protocol. Both cell lines and differentiation protocols were investigated regarding their ability to generate OCs and their inherent robustness and ease of use. First, both cell lines’ ability to remain undifferentiated while propagating in a feeder-free system was assessed. This was followed by evaluating mesodermal differentiation and characterization of hematopoietic progenitor cells produced under the differentiation strategy. Finally, OC yield and OC functionality based on resorptive activity, Cathepsin K and TRAP expression were assessed and compared.

## Methods

### iPSC culture

The OC proliferation and differentiation of two iPSC lines derived from distinct tissue origins were compared. MCND-TENS2, a peripheral blood mononuclear CD34^+^/CD38^−^ cell-derived iPSC line from a healthy donor (37) (received from NIH National Heart Lung Blood Institute, Bethesda, MD, USA; registered at https://hpscreg.eu/cell-line/RTIBDi001-A) was compared to GM28404*B, a fibroblast-derived iPSC line documented to originate from “apparently healthy individuals” (received from Coriell Institute Cell Repository, Camden, NJ, USA; registered at https://www.cellosaurus.org/CVCL_C0M4). Both cell lines were created using a Sendai virus reprogramming kit (37, 43). Upon defrosting, cells were cultured with mTeSR Plus (StemCell Technologies, Vancouver, Canada) on Cultrex basement membrane extract (R&D Systems, Minneapolis, USA) coated 6-well plates and incubated at 37°C and supplied with 5% CO_2_. 10 μM of Rho-associated, coiled-coil containing protein kinase (ROCK) inhibitor Y-276432 was used when defrosting to improve cell survival. Media changes were performed every second day. iPSCs were passaged at 70–80% confluency using 5 U/mL Dispase (StemCell Technologies, Vancouver, Canada).

### Mesodermal induction, hematopoietic differentiation, and osteoclast differentiation

In this study a cytokine defined differentiation protocol published by Rössler et al.(31) was compared to a commercially available differentiation kit (STEMdiff hematopoietic kit, StemCell Technologies, Vancouver, Canada), using the above-mentioned iPSC lines from different tissue origins. Both cell lines underwent mesodermal induction at passage 27.

EB differentiation according to Rössler et al. (31) was performed by creating a single cell suspension which was seeded in a round bottom ultra-low attachment 96-well plate at a cell density of 1.25×10^4^ cells in 100 μL of mTeSR Plus supplemented with 50 ng/mL human bone morphogenetic protein 4 (BMP4) (StemCell Technologies, Vancouver, Canada), 50 ng/mL human vascular endothelial growth factor-165 (VEGF165) (StemCell Technologies, Vancouver, Canada), 20 ng/mL human stem cell factor (SCF) (StemCell Technologies, Vancouver, Canada), and 10 μM ROCK inhibitor Y-27632 (StemCell Technologies, Vancouver, Canada). Subsequently, the 96-well plate was centrifuged for 3 min at 100 × g and half medium changes performed after one and two days. Four days after mesodermal induction, embryoid bodies were transferred to a 6-well plate and further differentiated in X-VIVO 15 Medium (Bioscience Lonza, Basel, Switzerland) supplemented with 2 mM Ultraglutamine (Bioscience Lonza, Basel, Switzerland), 55 μM 2-mercaptoethanol (ThermoFisher, Waltham, USA), 25 ng/mL human interleukin 3 (IL-3) (StemCell Technologies, Vancouver, Canada), 100 ng/mL human macrophage colony-stimulating factor (M-CSF) (StemCell Technologies, Vancouver, Canada). A full medium change of 3 mL was performed after 5 days. After 10 days of differentiation, floating suspension cells were harvested and used for further OC differentiation.

MB differentiation using STEMdiff hematopoietic kit (37) (StemCell Technologies, Vancouver, Canada) was performed according to manufacturer’s instruction. In short, 16–40 cell aggregates were seeded onto each 12-well plate coated with Cultrex basement membrane extract and treated for 3 days with medium A (containing bFGF, BMP4, VEGFA) (37) and consecutively for 10 days with medium B (containing bFGF, BMP4, VEGFA, SCF, Flt3L, TPO) (37). The floating suspension cells were harvested at the end of the period and used for further OC differentiation.

Following hematopoietic differentiation cells were transferred to 6-well plates at 2 × 10^5^ cells/cm^2^ and incubated in alpha-MEM (Bioscience Lonza, Basel, Switzerland) supplemented with 10% heat-inactivated fetal bovine serum (FBS) and 50 ng/mL human M-CSF for 3 days after which cells were incubated in alpha-MEM supplemented with 10% heat-inactivated FBS, 50 ng/mL human M-CSF and 70 ng/mL human RANKL (StemCell Technologies, Vancouver, Canada) for 7 days. Medium changes were performed every 2–3 days. OC differentiation was finished by a final treatment using alpha-MEM supplemented with 10% heat-inactivated FBS and 80 ng/mL human RANKL for 2 days.

### Enzymatic staining

To assess the degree of spontaneous differentiation during cell expansion, both iPSC lines were stained for alkaline phosphatase (ALP) (Abcam, Cambridge, UK) as a marker for pluripotency each time the cell lines were passaged. Additionally, ALP expression was assessed throughout differentiation. For this, cells were seeded onto 8-chamber slides, fixed at 70–80% confluency and stained according to manufacturer’s protocol. Tiled full well images were taken using an inverted widefield microscope (DMI6000, Leica, Wetzlar, Germany).

Tartrate-resistant acid phosphatase (TRAP) staining was performed after M-CSF matured hematopoietic cells were seeded onto a calcium-phosphate 24-well bone resorption assay plate (Cosmo Bio, Tokyo, Japan) in order to assess osteoclastogensis using a TRAP staining kit (Sigma-Aldrich, St. Louis, MO, USA). Staining was performed according to manufacturer’s instructions. In short, cells were fixed using 4% PFA, washed, staining mix added to the plate and incubated for 20 min at 37°C. OCs were then counterstained with methyl green nuclear stain (Sigma-Aldrich, St. Louis, MO, USA) for 10 minutes at room temperature. Image acquisition was performed using a Leica DMI6000 inverted microscope in phase-contrast mode and tiled full well images were analyzed for cell size and multi-nucleation using ImageJ.

### Immunocytochemistry

Immunocytochemistry (ICC) was performed after EB or MB induction in order to assess mesodermal differentiation and after termination of RANKL treatment in order to assess OCs.

After EB induction, cells were fixed with 4% paraformaldehyde (PFA) and blocked with PBS containing 10% normal goat serum (Abcam, Cambridge, UK), 0.3% Triton X-100 (Sigma-Aldrich, St. Louis, MO, USA), and 1% bovine serum albumin (BSA) (Sigma-Aldrich, St. Louis, MO, USA). After blocking, embryoid bodies were incubated overnight with antibodies against SOX1 (NL 493-conjugated) and Otx-2 (NL557-conjugated) to assess ectodermal differentiation, Brachyury (NL557-conjugated) and HAND1 (NL637-conjugated) to assess mesodermal differentiation or GATA-4 (NL493-conjugated) and SOX17 (NL637-conjugated) to assess endodermal differentiation (all germ-layer antibodies from R&D Systems, Minneapolis, USA) (Supplementary information, Table 1). All cells were counterstained for 15 min with 4′,6-diamidino-2-phenylindole (DAPI) (R&D Systems, Minneapolis, USA). Images were acquired using a Leica SP8X confocal laser scanning microscope (CLSM) and analyzed using ImageJ.

Following OC differentiation, cells were fixed with 4% PFA, permeabilized with Triton X-100 (ThermoFisher, Waltham, USA) for 30 min and blocked with normal goat serum for 60 min. Following permeabilization and blocking, OCs were stained with a primary anti-Cathepsin K antibody (Abcam, Cambridge, UK) which was succeeded by staining with an Alexa 647-conjugated secondary antibody (Abcam, Cambridge, UK) (Supplementary information, Table 1). Cells were then additionally stained with TRITC-conjugated Phalloidin and DAPI nuclear stain. Images were acquired and analyzed as mentioned above.

### Cell number and viability of hematopoietic cells

Following hematopoietic differentiation, floating suspension cells were collected and analyzed using a Countess 3 cell counter (ThermoFisher, Waltham, USA). Cell viability and cell number was measured after Trypan blue was added to testing samples.

### Flow cytometry

Hematopoietic differentiation was assessed by harvesting monocyte-like suspension cells, staining with LIVE/DEAD Fixable Violet stain (ThermoFisher, Waltham, USA) and blocking Fc receptors using TruStain FcX (Biolegend, San Diego, USA). This was followed by fixing cells with 4% PFA (Electron Microscopy Sciences, Hatfield, USA) and subsequent staining with primary antibodies (Supplementary information, Table 1) against CD34 (PE-Cy7-conjugated) (BD, Franklin Lakes, USA), CD43 (PerCp-Cy5.5-conjugated) (BD, Franklin Lakes, USA), CD45 (APC-conjugated) (BD, Franklin Lakes, USA), CD14 (BV711-conjugated) (BD, Franklin Lakes, USA), CD11b (PE-Cy5-conjugated) (Biolegend, San Diego, USA) and CD265/RANK (PE-conjugated) (R&D Systems, Minneapolis, USA), at 4°C for 1h in PBS supplemented with 0.09% (w/v) sodium azide (ThermoFisher, Waltham, USA) and 1% heat-inactivated FBS. Data acquisition and analysis was performed as mentioned above. Positive control and fluorescence compensation was performed with UltraComp eBeads (ThermoFisher, Waltham, USA). Gating was performed using isotype controls following singlets and live/dead gating. Data was acquired using a BD FACSymphony A3 (BD, Franklin Lakes, USA) and analyzed with FlowJo 10 (BD, Franklin Lakes, USA).

### Mineral resorption assay

In order to determine the mineral resorptive capacity of differentiated OCs, HPCs were plated onto a calcium-phosphate 24-well resorption assay plate and treated as mentioned above. After terminating OC differentiation, OCs were removed using 5% bleach and resorptive area was analyzed by taking tiled full well images as described above. OCs derived from human CD34^+^ PBMCs (healthy donor, received from Fred Hutch, Seattle) were used as positive control. In addition to image acquisition using the phase-contrast mode, images for resorption area quantification were taken using the yellow channel in fluorescence mode in order to facilitate differentiation between resorbed and un-resorbed areas in ImageJ.

### Analysis of gene expression

RNA extraction and isolation throughout the differentiation process for both iPSC lines using either the EB or the MB method was performed using a RNeasy kit (Qiagen, Hilden, Germany) according to the manufacturer’s protocol. In short, a lysis buffer was added to the cells and lysed mechanically using a 20G needle and syringe. Following RNA extraction RNA was isolated and purified using spin-columns. RNA concentration was measured using a NanoDrop (ThermoFisher, Waltham, USA). A total of 250 ng of RNA was used to generate complementary DNA (cDNA) using Omniscript (Qiagen, Hilden, Germany) at 37°C for 1 hour. The cDNA was then used to determine the expression of POU5F1, CSF1R, TNFRSF11A, NFATC1, CA2, MMP9 using a TaqMan quantitative reverse transcription PCR (qRT-PCR) (Supplementary information, Table 2). Gene expression levels were normalized to 18s ribosomal RNA levels and calculated using the ΔΔCt method to determine fold gene expression throughout the differentiation process.

## Statistical analysis

Statistical analysis was carried out using GraphPad Prism 9. Data are shown as means ± SD. Statistical significance was assessed using Tukey’s multiple comparison post-hoc test unless indicated otherwise in individual experiments. An adjusted p-value of *p* < 0.05 was considered to be statistically significant.

## Results

### iPSCs remain undifferentiated during propagation and retain ALP expression in centrally located cells throughout differentiation

**Figure 1:**
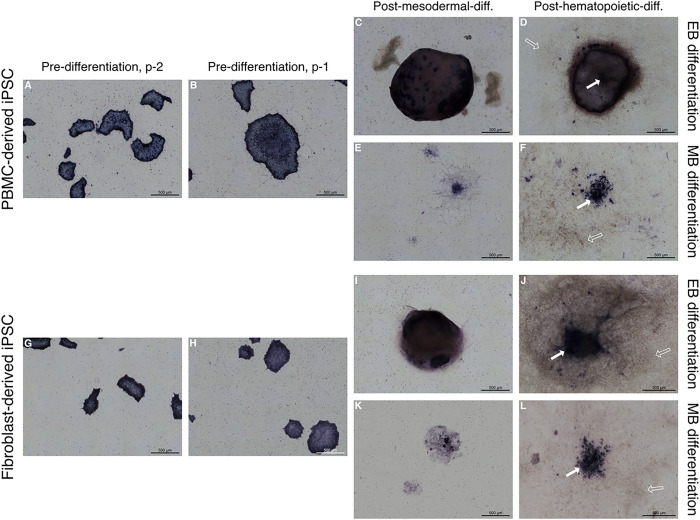
Assessment of alkaline phosphatase (ALP) expression in iPSC lines prior and throughout mesodermal and hematopoietic differentiation. **A-L** Representative images of 3 replicates display ALP-stained colonies and cell-forming complexes of a PBMC-derived cell line (A-F) and a fibroblast-derived cell line (G-L), showing high expression of ALP while expanding (A, B, G, H). Following mesodermal and hematopoietic differentiation either according to the embryoid body-based (C, D, I, J) or monolayer-based protocol (E, F, K, L), cell-forming complexes retained ALP expression, especially in centrally located cells (solid arrows). An abundance of ALP negative cells can be observed following hematopoietic differentiation (empty arrows). Scale bar = 500 μm.

ALP staining was performed for both PBMC and fibroblast-derived iPSC lines and used as a stem cell marker in order to monitor spontaneous differentiation and confirm stemness of the described iPSC lines prior to mesodermal induction (Fig. 1). Both cell lines showed a consistent ALP expression pattern throughout cell propagation while the cell colony peripheries stained more intensively as has been previously described elsewhere (44) (Fig. 1A, B, G, H). Both cell lines retained a certain degree of ALP expression following mesodermal differentiation under either using the EB differentiation (Fig. 1C, D, I, J) or MB differentiation protocol (Fig. 1E, F, K, L). There is a higher degree of ALP expression can be seen in fibroblast-derived iPSCs differentiated under the MB protocol (Fig. 1K) in comparison to the PBMC-derived iPSCs (Fig. 1E). Additionally, a higher degree of variability in size and distribution was observed in MB cell forming complexes (data not shown). Following hematopoietic differentiation, cell-forming complexes still retained a level of ALP expression in the most centrally located cells within the cell forming complex (arrows in Fig. 1D, F, J, L). Cell-forming complexes of the PBMC-derived iPSCs differentiated under the EB protocol (Fig. 1D) appeared to maintain their structure to a higher degree compared to the fibroblast-derived, EB cell-forming complexes (Fig. 1J). As expected, an abundance of ALP negative cells was observed following hematopoietic differentiation in both differentiation protocols (empty arrows in Fig. 1D, F, J, L).

### EB cell forming complexes display a higher degree of organization compared to MB cell forming complexes

**Figure 2:**
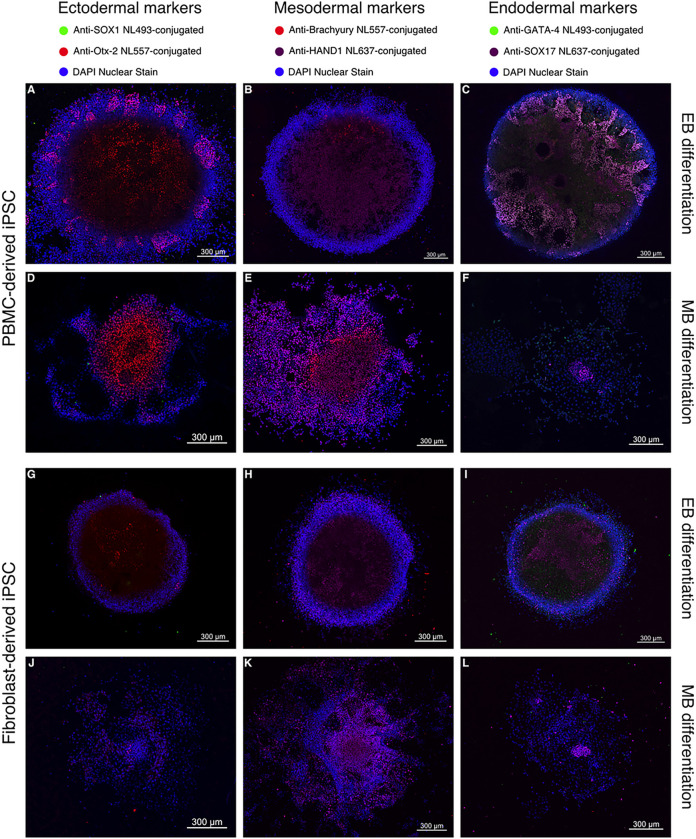
Analysis of cell-forming complexes following mesodermal differentiation using CLSM. **A-L** Representative images of 3 replicates show PBMC-derived iPSCs (A-F) or fibroblast-derived iPSCs (G-L) differentiated either according to an embryoid body-based (EB) protocol (A, B, C, G, H, I) or a monolayer-based (MB) protocol (D, E, F, J, K, L) and stained for ectodermal (A, D, G, J), mesodermal (B, E, H, K), and endodermal markers (C, F, I, L). Expression patterns of cell-forming complexes of PBMC-derived iPSCs demonstrate a higher degree of organization compared to complexes formed by fibroblast-derived iPSCs. Scale bar = 300 μm

Immunocytochemistry in conjunction with confocal microscopy of the cell-forming complexes was performed to assess the differentiation process following mesodermal induction (Fig. 2). As depicted, all cell-forming complexes, originating either from PBMC-derived iPSCs or fibroblast-derived iPSCs and differentiated either according to EB or MB differentiation protocols expressed markers for all three germ layers. In comparison to the fibroblast-derived cell line (Fig. 2G–L), the PBMC-derived cell line showed a higher degree of organization based on the expression of the ectodermal, mesodermal, and endodermal transcription factors under both EB (Fig. 2A–C) and MB differentiation protocols (Fig. 2D–F). Both cell lines and both differentiation protocols showed a higher expression of the ectodermal marker Otx-2 compared to SOX1 (Fig. 2A, D, G, J). SOX17, an endodermal marker, was consistently expressed centrally within cell-forming complexes differentiated according to the MB protocol in both iPSC lines (Fig. 2F and L). EB cell-forming complexes from PBMC-derived iPSCs expressed the mesodermal marker Brachyury (Fig. 2B) while EB cell-forming complexes originating from the fibroblast-derived cell line expressed low to no Brachyury (Fig. 2H).

### EB differentiation gives rise to later stage hematopoietic cells compared to MB differentiation

Following hematopoietic differentiation, the suspension cell population that arose from the cell-forming complexes was harvested and analyzed for quantity and cell viability (Supplemental Fig. 1). More cells were harvested from cell-forming complexes differentiated according to the EB protocol (Supplemental Fig. 1A) compared to the MB protocol independent of the iPSC lines used in this study.

**Figure 3:**
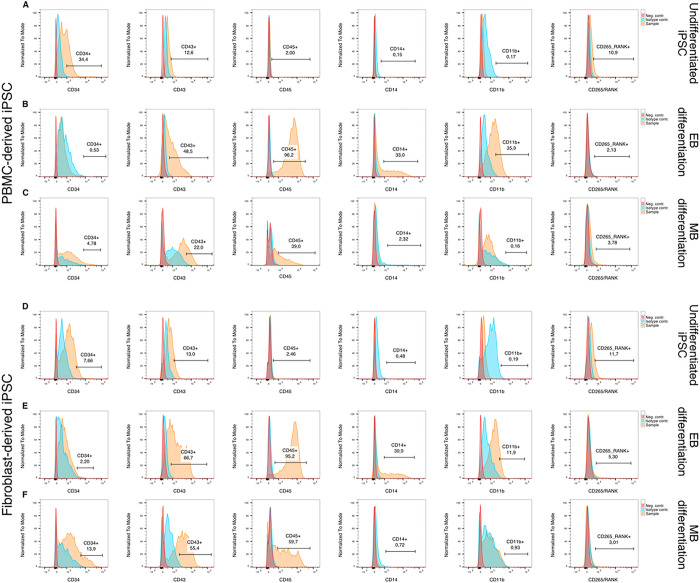
Assessment of hematopoietic cells using flow cytometry. **A, D** Undifferentiated iPSCs were used as a reference for marker expression. **B, C, E, F** Hematopoietic cells differentiated according to the embryoid body-based (EB) protocol show a higher expression of later hematopoietic markers CD43 and CD45 (B, E) in comparison to monolayer-based (MB) differentiated cells (C, F). Additionally, monocyte markers CD14 and CD11b were elevated in the EB group (B, E). No differences in RANK expression could be observed between either differentiation protocol.

In order to further characterize the harvested floating cell population, flow cytometry was performed using hematopoietic and monocytic markers (Fig. 3). Additionally, cells were stained with an anti-RANK antibody to investigate whether an early difference in RANK expression can account for differences in OC activity between cell lines and differentiation protocols.

Undifferentiated iPSCs, used as negative control (Fig. 3A and D), displayed a CD34^+^ sub-population which was larger in the PBMC-derived iPSC line compared to the fibroblast-derived iPSC line. Additionally, both undifferentiated iPSCs populations yielded a small fraction of a CD265^+^ sub-population.

EB differentiated cells generated larger CD43^+^ and CD45^+^ populations (Fig. 3B and D) compared to MB differentiated cells while MB differentiation resulted in larger CD34^+^ populations (Fig. 3C and F). Additionally, floating hematopoietic cells differentiated according to the EB protocol had a larger population of monocytes (CD14^+^ and CD11b^+^ cells) for both iPSC lines (Fig. 3B and E). No differences were observed in RANK expression between both differentiation protocols or cell lines.

### EB differentiation gives rise to bona fide OCs

**Figure 4:**
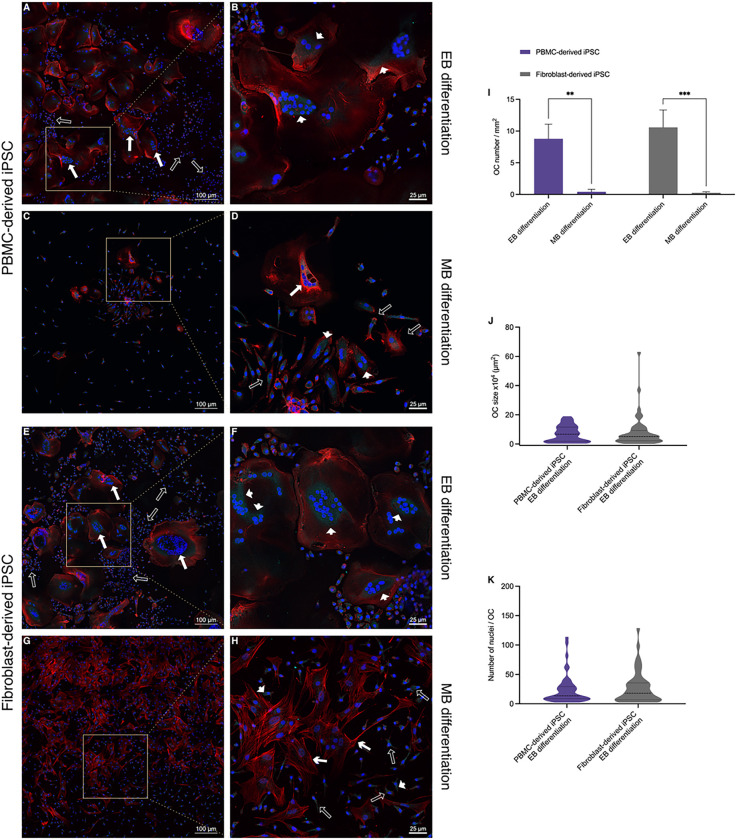
Morphology assessment of cells following osteoclast differentiation using CLSM. **A-H** Representative CLSM images of hematopoietic cells from an PBMC-derived iPSC line (A-D) or a fibroblast-derived iPSC line (E-H) which had been differentiated either according to an embryoid body-based (EB) (A, B, E, F) or a monolayer-based (MB) protocol (C, D, G, H) were further subjected to osteoclast differentiation and stained for Cathepsin K (turquoise), F-actin (red) and counterstained with DAPI nuclear stain (blue). Cells differentiated according to the EB protocol showed large multinucleated polykaryons (solid arrows in A, E) which also demonstrated Cathepsin K expression (arrow tips in B, F). MB differentiated cells on the other hand showed a low number of cells with up to 5 nuclei (solid arrow in D) in the PBMC-derived iPSC line and cells with a stellar-like morphology in the fibroblast-iPSC line (chevron arrows in H). A limited number of cells expressing Cathepsin K can be seen in both groups (arrow heads in D, H). Mononuclear cells with some degree of Cathepsin K expression can be seen throughout all groups (empty arrows in A, D, E, H). **I-K**Image quantitation shows a significant difference in osteoclast number (3 or more nuclei) between the EB and the MB protocols (I). No significant differences were observed in osteoclast size or number of nuclei when the EB protocol was used with the different iPSC lines (J, K). Scale bars: A, C, E, G = 100 μm, B, D, F, H = 25 μm. Statistics are based on ANOVA followed by Tukey’s multiple comparison post-hoc test ((I) *n* = 3 well replicates, (J, K) *n*= 50 analyzed cells, ** *p* < 0.01, *** *p* < 0.001).

Following OC differentiation, cells were stained for Cathepsin K (turquoise) and F-actin (red) and counterstained with nuclear DAPI stain (blue) (Fig. 4). Both fibroblast and PBMC-derived iPSC lines showed multiple large spread-out multinucleated cells when differentiated according to the EB protocol (Fig. 4A, B, E, F). Several large polykaryons with up to 100 nuclei were observed in both groups (solid arrows in Fig. 4A and E), while those derived from the fibroblast iPSC line appeared to exhibit more Cathepsin K (arrow heads in Fig. 4F) compared to the PBMC-derived cell line (arrow heads in Fig. 4B). Both cell lines had the strongest signals for Cathepsin K in proximity to nuclei clusters. Additionally, mononuclear cells with varying degrees of Cathepsin K expression were interspersed with the large multinucleated cells (empty arrows in Fig. 4A and E).

The relative homogeneity of the two cell lines differentiated according to the EB protocol (Fig. 4A, B, E, F) was contrasted by the distinct appearances of PBMC- and fibroblast-derived cell lines differentiated according to the MB protocol (Fig. 4C, D, G, H). Similar observations were made between multiple experiments. First, PBMC-derived iPSCs displayed only few multinucleated cells with no more than 5 nuclei per cell (solid arrow in Fig. 4D). Cathepsin K expression was observed in some of the mono and multinucleated cells (arrow heads in Fig. 4D). While some cells showed a spread-out morphology, most cells were mononuclear and had a stellar or spindle-like appearance (empty arrows in Fig. 4D). In contrast to the PBMC-derived cell line, the fibroblast-derived iPSC line (Fig. 4H) exhibited mononuclear cells with a distinct stellar-like phenotype following OC differentiation. While most of these stellar-like cells appeared to express little to no Cathepsin K (arrow heads in Fig. 4H), a well-formed F-actin cytoskeletal structure was visible following visualization of F-actin with TRITC-conjugated Phalloidin (chevron arrows in Fig. 4H). Small mononuclear cells with varying degrees of Cathepsin K expression were also seen in this group (empty arrows in Fig. 4H). Image quantitation confirmed the significant differences between both protocols (Fig. 4I) while demonstrating no significant differences in osteoclasts differentiated according to the EB protocol (Fig. 4J and K) using either the PBMC or fibroblast-derived cell line.

**Figure 5:**
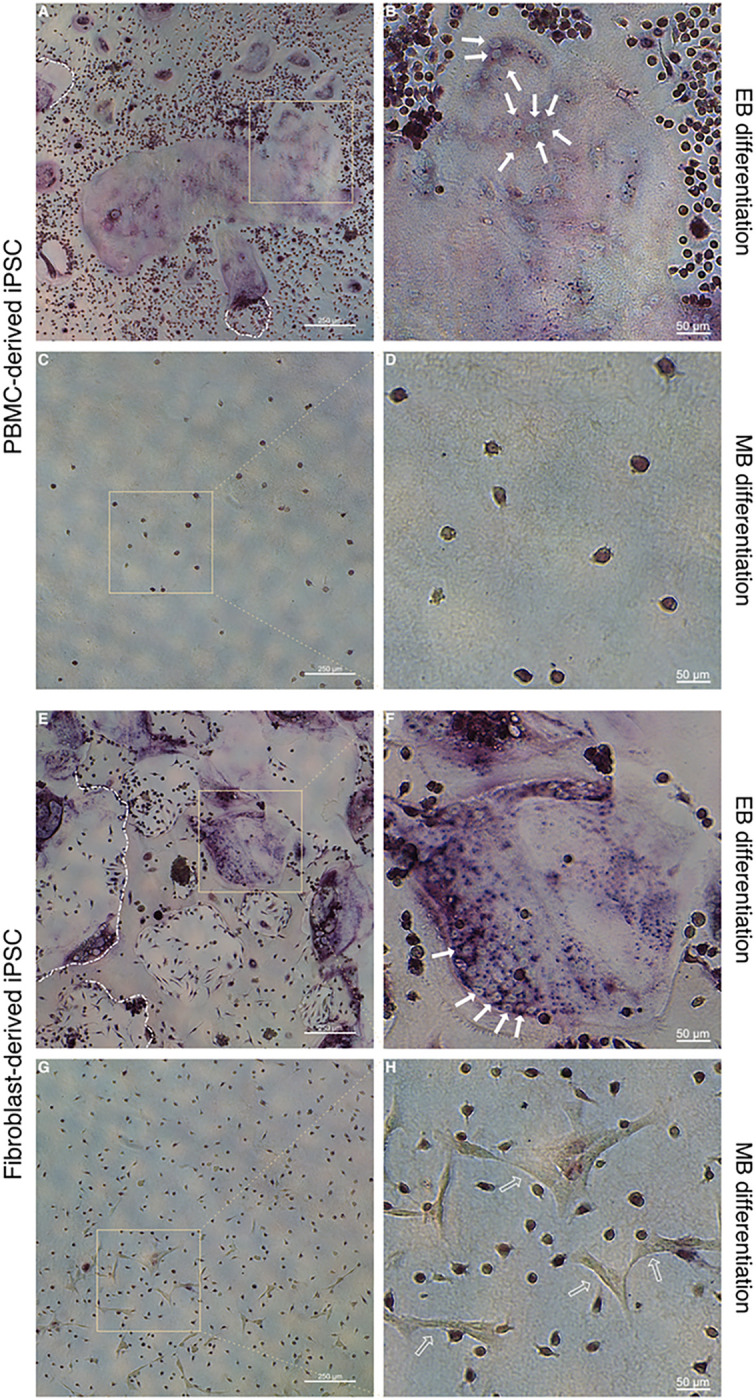
TRAP staining of cells following osteoclast differentiation in conjunction with methyl green nuclear counterstain. **A-H** Hematopoietic cells from an PBMC-derived iPSC line (A-D) or a fibroblast-derived iPSC line (E-H) which had been differentiated either according to an embryoid body-based (EB) (A, B, E, F) or a monolayer-based (MB) protocol (C, D, G, H) were seeded onto calcium-phosphate coated wells and further subjected to osteoclast differentiation conditions. Representative images of cells derived from EB protocols show large TRAP positive cells (A, B, E, F) with multiple nuclei (solid arrows in B, F). Resorption pits are also visible in both groups (white dashed lines in A, E). Cells derived from the MB protocol did not give rise to osteoclasts. Cells with a stellar-like cell morphology can be seen in the fibroblast-derived iPSC MB differentiation group (empty arrows in H). Scale bars: A, C, E, G = 250 μm, B, D, F, H = 50 μm.

As presented in Fig. 5, both iPSC lines differentiated according to the EB protocol generated large, multinucleated OCs following OC differentiation (Fig. 5A, B, E, F) as determined by TRAP and methyl green nuclear counterstaining (solid arrows in Fig. 5B and F). TRAP is commonly used to stain OCs, albeit also expressed in leukocytes as further discussed below. Resorption pits are visible in both groups (borders outlined using white dashed lines in Fig. 5A and E). OCs differentiated from the fibroblast-derived cell line under the EB protocol were larger, more numerous and stained more intensively for TRAP compared to the PBMC-derived cell line. Both cell lines differentiated according to the MB protocol (Fig. 5C, D, G, H) show lightly stained TRAP positive mononuclear cells. A lower cell density of mononuclear cells is observed for the PBMC-derived cell line (Fig. 5D) in comparison to the fibroblast-derived cell line (Fig. 5G and H). Analogous to the findings in Fig. 4G and H, Fig. 5H shows again TRAP^−^ cells with a stellar-like phenotype (empty arrows in Fig. 5H).

### Overall, assessment of cell morphology as well as expression of Cathepsin K and TRAP demonstrate the differentiation of human OCs when using the EB protocol.

**Figure 6:**
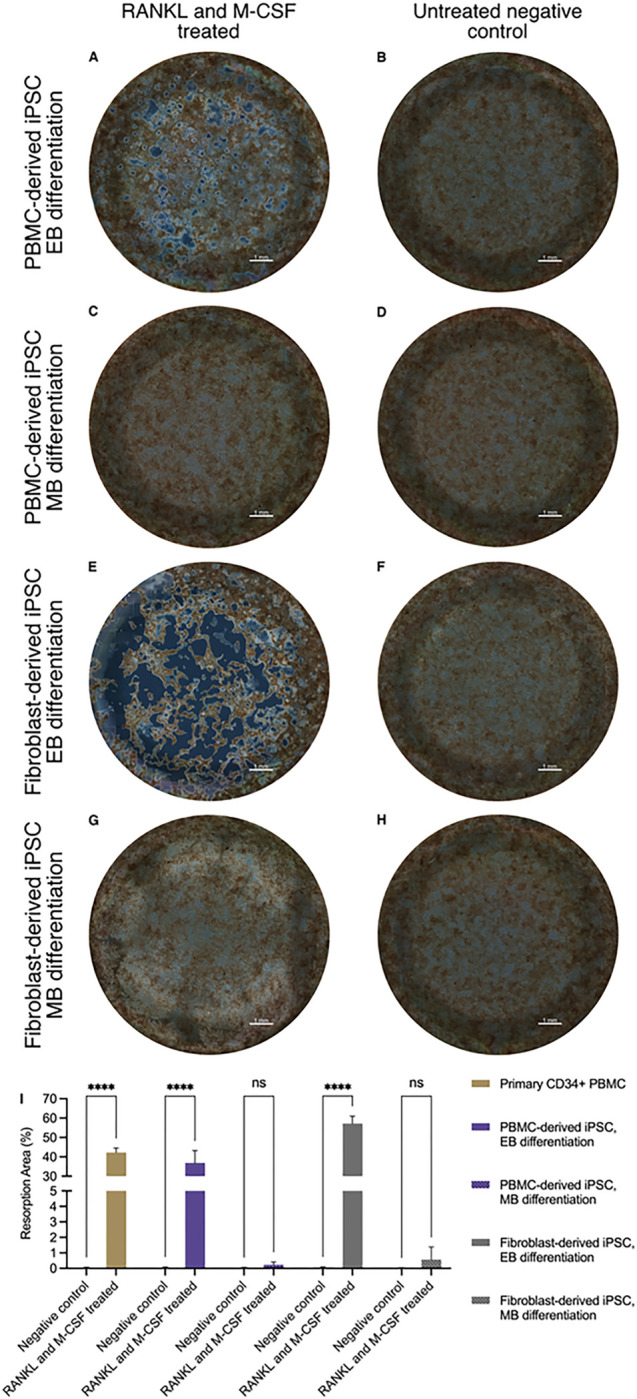
Assessment and quantification of mineral resorption activity of osteoclasts. **A-H** Following hematopoietic differentiation of PBMC-derived iPSCs (A-D) or fibroblast-derived iPSCs (E-H), either according to an embryoid body based (EB) (A, B, E, F) or a monolayer-based (MB) protocol (C, D, G, H), hematopoietic cells were matured with M-CSF and differentiated to OCs with RANKL on calcium-phosphate coated wells. Osteoclasts differentiated from PBMC-derived iPSCs using the EB protocol showed clearly visible resorption pits on tiled full-well images acquired with a widefield microscope in phase-contrast mode (A) in comparison to undifferentiated negative controls (B). Similarly, osteoclasts from fibroblast-derived iPSCs differentiated with the EB protocol also showed clearly visible pits, albeit the total resorption area appears much larger (E) compared to the negative control (F). In comparison, cells differentiated according to the MB protocol did not show visible resorption pits for either cell line (C, G) when compared to negative controls (D, H). Scale bar = 1 mm. **I** Image quantification demonstrates comparable resorption levels of PBMC-derived iPSC osteoclasts differentiated according to the EB protocol to osteoclasts differentiated from primary CD34^+^ PBMCs. The highest level of mineral resorption was seen in osteoclasts differentiated from the fibroblast-derived iPSC line using the EB protocol. Quantification confirms the absence of mineral resorption in cells differentiated according to the MB protocol for either iPSC line. Statistics are based on ANOVA followed by Tukey’s multiple comparison post-hoc test (*n* = 3 well replicates, **** *p* < 0.0001).

OC resorptive activity was assessed by quantifying the resorption area on calcium-phosphate coated wells (Fig. 6). Wells with OCs differentiated according to the EB protocol showed clearly visible resorption pits for both PBMC-derived (Fig. 6A) and fibroblast-derived cell lines (Fig. 6E). However, the quantified area of resorption pits created by OCs from the fibroblast-derived iPSC line was significantly higher than from the PBMC-derived cell line (36.9% vs 57.2%, p < 0.05). In contrast, wells with OCs differentiated according to the MB protocol (Fig. 6C and G) showed almost no visible resorption pits.

**Figure 7:**
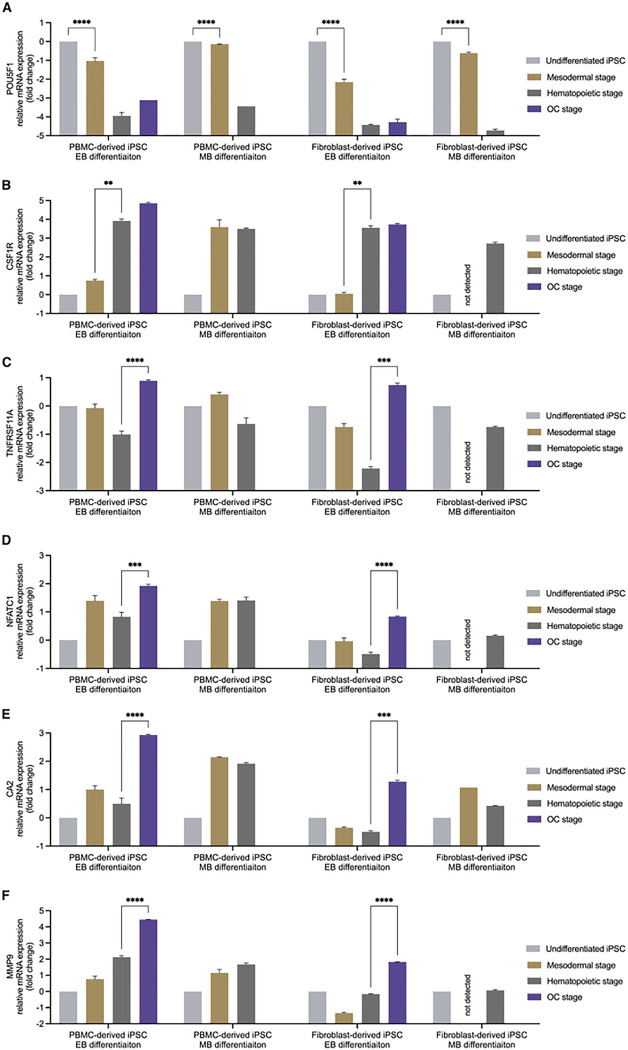
Relative gene expression of iPSCs throughout the differentiation process. Gene expression of POU5F1 decreased in all groups significantly from the mesodermal to the hematopoietic stage (A). CSF1R increased significantly after the mesodermal in relation to the hematopoietic stage in the embryoid body-based (EB) differentiation protocol (B) independent of iPSC line. Monolayer-based (MB) differentiation showed either no significant increase or an initial Ct value below the detection threshold. Osteoclast markers all showed a significant increase in the EB protocol for both iPSC lines while MB differentiation did not yield sufficient RNA for analyses (C-F). Statistics are based on multiple comparisons using the Holm-Šídák method (*n* = 3 replicates, except POU5F1: OC stage of PBMC-derived iPSC EB differentiation and hematopoietic stage of PBMC-derived iPSC MB differentiation, as well as CA2: mesodermal stage of fibroblast-derived iPSC MB differentiation, where all replicates were close to, while one replicate was below the detection threshold, * *p* < 0.05, ** *p* < 0.01, *** *p* < 0.001, **** *p* < 0.0001).

To further assess gene expression throughout the differentiation process, qRT-PCR was performed (Fig. 7) using a stemness marker (Fig. 7A), an osteoclast precursor marker (Fig. 7B) and osteoclast markers (Fig. 7C, D, E, F). The expression of POU5F1 (Oct4) was significantly reduced throughout the differentiation process in all groups (Fig. 7A). However, it can be noted that iPSCs differentiated according to the EB protocol showed a larger decrease compared to the MB differentiation protocol. CSF1R (M-CSFR) showed a significant increase during transition from the mesodermal to the hematopoietic stage during EB differentiation (Fig. 7B). PBMC-derived iPSCs differentiated according to the MB protocol showed no increase in CSF1R. Ct values for CSF1R within the fibroblast-derived iPSC line were below the detection threshold. OC markers RANK (Fig. 7C) and NFATC1 (Fig. 7D) showed a significant increase in expression in the OC stage compared to the previous hematopoietic stage when differentiated according to the EB protocol. This increase was seen in both iPSC lines. Additionally, markers associated with resorptive activity, CA2 (Fig. 7E) and MMP9 (Fig. 7F) were also significantly elevated in the OC stage when differentiated using the EB protocol.

## Discussion

Several hematopoietic differentiation protocols have been published. However, it remains unclear which protocol or differentiation method is favorable for osteoclastogenesis. Here, we compare an EB to a MB differentiation protocol using iPSC lines from different tissue origins. We found that EB differentiation yielded bona fide osteoclasts independent of tissue origin of the iPSC line used while MB differentiation led to hematopoietic cells of earlier stages which did not subsequently differentiate into OCs.

Differentiation of iPSCs into OCs requires a complex series of events. While the entire differentiation process can be divided into three major steps of mesodermal, hematopoietic and OC differentiation, each step can be further subdivided into steps that are recapitulated in ontogenesis. Hematopoietic differentiation has been shown to require stages of mesodermal induction, hematoendothelial specification, vascular arterialization, endothelial-to-hematopoietic transition, and hematopoietic maturation (40, 45). Both EB and MB approaches have previously been reported to recapitulate these steps to varying degrees (37, 46–52).

A prolonged expression of pluripotency markers has been described elsewhere during EB formations (53, 54), similar to the retained ALP expression in the center of cell-forming complexes of both EB and MB protocols even after hematopoietic differentiation.

EB differentiation has been reported to simulate early stages of post-implantation development by exhibiting ectodermal, mesodermal, and endodermal germ layers (40). Accordingly, embryoid bodies showed expression patterns for all three germ layers while differences in the degree of their expression and spatial organization existed between the two iPSC lines from different tissue origins. In contrast to the EB differentiation approach, MB differentiation showed similar, yet not as spatially organized expression patterns of markers for the three germ layers.

Cell forming complexes, either EB or MB, have the potential to influence and alter hematopoietic cell production. This occurs through cell-cell interactions, extracellular matrix (ECM) interactions, and cytokine signaling from cell niches (53, 55). The right microenvironment therefore appears to be critical for successful hematopoietic differentiation (53, 56–58). For example, Sturgeon et al. showed that activation of Wnt–β-catenin during mesodermal specification can determine whether cells will undergo a definitive hematopoietic program (59–61) or a primitive program.

It has been shown that endogenous Wnt–β-catenin signaling is more active in EB (or 3D) cell-forming complexes over MB (or 2D) cell forming complexes (62). It could be hypothesized, that due to the larger relative surface area of a monolayer compared to a sphere, the MB cell-forming complexes cannot maintain their own microenvironment as well as EB complexes and are more susceptible or dependent on the exogenous cytokine concentrations and composition. Differentiation outcomes of embryoid bodies have also been reported to be highly variable based on their size and quality (40), which could also be explained by the same hypothesis.

Despite variability in the size of embryoid bodies, EB differentiation yielded more hematopoietic cells with a higher cell viability compared to MB differentiation. Even though MB differentiation in conjunction with pre-made differentiation media is less work intensive and a significant improvement in usability, this method demonstrated more variability from well to well with respect to numbers of iPSC colonies per well, colony size and their distribution within the well.

Nevertheless, both differentiation approaches gave rise to hematopoietic cells. Even though some variation between iPSC lines could be observed, MB cells showed a larger CD34^+^ population for both iPSC lines, whereas the EB differentiated cell populations were comprised of large portions of CD43^+^ and CD45^+^ cells. Both CD43 and CD45 appear ontogenetically after CD34, and CD45 is associated with progressive myeloid commitment (63–65). Additionally, EB differentiated cells comprised a larger portion of CD11b^+^ and CD14^+^ monocytes, suggesting an overall further downstream position compared to MB hematopoietic cells. These findings were consistent with findings by Rössler et al., who showed similar population sizes of CD14^+^ and CD11b^+^ cells using EB differentiation, albeit being slightly higher than our populations (31). Ruiz et al. on the other hand did not assess CD14 and CD11b marker expression, showed however a larger CD43^+^ population compared to the CD45^+^ population after 10 days of differentiation when differentiated according to the MB protocol (37). This was comparable to our findings, showing a smaller CD45^+^ population for the MB protocol compared to the EB protocol. Nevertheless, a prolongation of treatment with the hematopoietic differentiation medium can increase the CD45^+^ portion (37) when differentiating hematopoietic cells according to the MB protocol using the STEMdiff hematopoietic kit.

While few studies have focused on in depth phenotyping of OC precursors (66) the traditional view depicts a multipotent progenitor (MPP) that is able to give rise to a common myeloid progenitor (CMP) and a common lymphoid progenitor (CLP). The CMP in turn gives rise to the megakaryocyte/erythrocyte progenitor (MEP) and granulocyte/macrophage progenitor (GMP). GMPs differentiate into granulocytes, monocytes, macrophages, dendritic cells, and OCs (66–71). GMPs are capable of giving rise to CD11b^+^ and CD14^+^ monocytes which are well known to be able to differentiate into OCs (72–76). However, CD14^−^ cells (75, 77) and a CD11b^−^CD14^−^CD115^+^ (CD115 ≙ CSFR1/c-FMS) population have also been shown to possess high osteoclastic potential (78). Whether or not CD14^−^ cells obligatorily pass through a CD14^+^ stage in order to differentiate into OCs remains to be seen. Recent work has shown that not only GMPs, but also a multilymphoid progenitor (MLP) is capable of giving rise to macrophages and dendritic cells in addition to lymphoid cells, while being devoid of a megakaryocyte/erythroid potential (68, 79, 80). Downstream of the GMP or MLP a CD11b^−^CD34^+^c-KIT^+^FLT3^+^IL3Rα^high^ population was identified as a common macrophage, OC and dendritic cell progenitor that was mRNA positive for CSFR1, did however not show any presence of CSFR1 at the cell surface (66). Trajectory analysis of an OC population using single cell RNA sequencing (scRNA-seq) shows a differentiation path beginning at a CD14^+^ stage, followed by a dendritic cell and macrophage stage before reaching a more active OC type, expressing increased levels of Cathepsin K and V-ATPase subunit D 2 (81). Early stage (c-Kit^+^, c-Fms^+^, Mac-1^dull^ and RANK^−^) and late-stage OC precursors (c-Kit^−^, c-Fms^+^, Mac-1^+^ and RANK^+^) have been described (82, 83) that are driven towards mature OCs by a wide range of factors (84–90). Ultimately, a mononuclear OC fuses with adjacent mononuclear OCs. This is regulated by proteins such as dendritic cell-specific transmembrane protein (DC-STAMP), osteoclast stimulatory transmembrane protein (OC-STAMP) and syncytin-B (91–93), to form a large multinucleated cell. These mature OCs are capable of mineral and bone resorption (94) and show an increase in expression of proteins related to the resorption process such as V-ATPase subunit D 2, carbonic anhydrase 2, Cathepsin K, and MMP9 (69, 81, 95–97).

We showed that iPSCs differentiated according to the EB protocol gave rise to large multinucleated cells for both PBMC- and fibroblast-derived iPSC lines. qRT-PCR results showed a significant increase in expression of TNFRSF11A, NFATC1, CA2 and MMP9, supporting their identity as OCs. Finally, TRAP expression, Cathepsin K expression, and mineral resorptive capacity clearly identify the differentiated multinucleated cells as bona fide OCs.

iPSCs differentiated using the MB method only gave off a very limited number of multinucleated cells with up to 5 nuclei for the PBMC-derived iPSC line, and no multinucleated, yet stellar-shaped appearing cells for the fibroblast-derived iPSC cell line. Both iPSC lines differentiated according to the MB protocol did not show mineral resorptive capacities. We hypothesize, that since the MB differentiation process gave rise to ontogenetically earlier HPCs compared to the EB method, MB cells might not have reached the OC stage in the same differentiation time span as compared to cells differentiated using the EB protocol and might have remained in earlier stages or side trajectories such as macrophages or dendritic cells.

Both EB or MB differentiated cells displayed TRAP^+^ mononuclear cells that might be presumed to be mononuclear OCs or OC precursors. However, TRAP expression is not specific to OCs but also present in immune cells such as macrophages and dendritic cells (DCs) (98–100). Isoform 5b has been found to be predominantly secreted by OCs (101) which could be used to further define mononuclear cells. However, single cell methodology such as scRNA-seq might offer the best possibility to get an overall picture of sub-populations and differentiation trajectories within the different groups (102).

Differences based on tissue origin of iPSC lines were observed regarding cell growth as fibroblast-derived iPSCs showed higher proliferation rates during propagation (data not shown). Fibroblast-derived iPSCs also gave off more hematopoietic cells compared with PBMC-derived iPSCs. Nevertheless, both cell lines could be successfully differentiated into OCs independent of their tissue origin. This is in line with research showing iPSC differentiation potential to be largely independent of cell type origin (103, 104), while minor differences have been attributed to epigenetic memory persisting even after reprogramming to iPSCs (105–107). On average, PBMC-derived iPSCs gave off 2289 OCs per embryoid body, while fibroblast-derived iPSCs gave off 5505 OCs per embryoid body.

Limitations of this study consist mainly in the use of a commercially available differentiation kit for MB differentiation, as cytokine composition and concentration are not fully disclosed and thus, findings in this study may be attributed in part to differing cytokines and not just on the 3D shape of EB cell-forming complexes over the 2D one in MB cell-forming complexes. Nevertheless, commercially available kits have its place and also require comparison and validation of established methods.

Regarding reproducibility, one additional limitation consists in the usage of FBS for terminal OC differentiation which can result in batch-to-batch variability (108) as our lab has noted difference in osteoclastogenesis when using exosome-free FBS over standard/untreated FBS (unpublished data). In view of clinical translation, a fully defined differentiation process without the use of FBS for terminal OC differentiation will be required (109).

Finally, the scope of this study did not reveal the exact subtype of OCs that was differentiated. Many different subtypes of OCs such as chrondroclasts, odontoclasts, septoclasts, vascular-associated OCs have been described by other authors (110–113). Single cell transcriptomics could offer also in this regard a deeper insight into differentiation trajectories and cell subtypes.

## Conclusion

The outcome of this study demonstrates the successful differentiation of OCs from iPSCs in conjunction with an EB differentiation method. In contrast, a MB differentiation method that used a commercially available hematopoietic differentiation kit did not yield OCs. Presence of bona fide OCs was validated using osteoclast marker expression and determining the mineral resorptive activity. The differentiation process was continually evaluated following mesodermal induction, hematopoietic differentiation and after terminal OC differentiation for both EB and MB approaches using iPSCs from two distinct tissue origins. No differences were observed regarding OC differentiation between a PBMC- and a fibroblast-derived iPSC line used in this study.

## Data Availability

The datasets supporting the conclusions of this article are available in the Dryad repository, [unique persistent identifier and hyperlink to dataset(s) in http:// format will be provided upon acceptance as Dryad requires a manuscript number which is generated upon submission].

## Abbreviations

ALP: Alkaline phosphatase
ANOVA: Analysis of variance
bFGF: Basic fibroblast growth factor
BMP4: Bone morphogenetic protein 4
BSA: Bovine serum albumin
CA2: Carbonic anhydrase 2
CD: Cluster of differentiation
cDNA: Complementary DNA
CID: Chemical inducer of dimerization
CLP: Common lymphoid progenitor
CLSM: Confocal laser scanning microscope
CMP: Common myeloid progenitor
CSF1R: Colony stimulating factor 1 receptor
CTSK: Cathepsin K
DAPI: 4′,6-diamidino-2-phenylindole
DC-STAMP: Dendritic cell-specific transmembrane protein
EB: Embryoid body-based
ECM: Extracellular matrix
FBS: Fetal bovine serum
Flt3L: FMS-like tyrosine kinase 3 ligand
GMP: Granulocyte/macrophage progenitor
HPCs: Hematopoietic progenitor cells
ICC: Immunocytochemistry
IL-3: Interleukin 3
iPSC: Induced pluripotent stem cells
iRANK: Engineered myeloid precursors with a conditional receptor activator of nuclear factor kappa-Β intracellular domain
M-CSF: Macrophage colony-stimulating factor
MB: Monolayer-based
MEM: Minimum essential medium
MEP: Megakaryocyte/erythrocyte progenitor
MLP: Multilymphoid progenitor
MMP9: Matrix metallopeptidase 9
MPP: Multipotent progenitor
NFATC1: Nuclear factor of activated T cells 1
OC: Osteoclasts
OC-STAMP: Osteoclast stimulatory transmembrane protein
OPG: Osteoprotegerin
OSCAR: Osteoclast-associated receptor
PBMCs: Peripheral blood mononuclear cell
PBS: Phosphate buffered saline
PFA: Paraformaldehyde
qRT-PCR: Quantitative reverse transcription polymerase chain reaction
RANK: Receptor activator of nuclear κB
RANKL: Receptor activator of nuclear κB ligand
ROCK: Rho-associated coiled-coil containing protein kinase
SCF: Stem cell factor
scRNAseq: Single cell RNA sequencing
TRAP: Tartrate-resistant acid phosphatase
